# Development and Validation of Multiple Equations for Low-Density Lipoprotein and Apolipoprotein B in Korean Patients Visiting Local Clinics and Hospitals

**DOI:** 10.3390/nu15122786

**Published:** 2023-06-17

**Authors:** Rihwa Choi, Sang Gon Lee, Eun Hee Lee

**Affiliations:** 1 Department of Laboratory Medicine, Green Cross Laboratories, Yongin 16924, Republic of Korea; pirate0720@naver.com; 2 Department of Laboratory Medicine and Genetics, Samsung Medical Center, Sungkyunkwan University School of Medicine, Seoul 06351, Republic of Korea; 3 Green Cross Laboratories, Yongin 16924, Republic of Korea

**Keywords:** apolipoprotein B, ApoB, dyslipidemia, utilization, translation, equation, Korea

## Abstract

We investigated the utilization of apolipoprotein B (ApoB), an independent risk factor for cardiovascular disease, and developed and validated a translational equation for calculating low-density lipoprotein cholesterol (LDL-C) in the Korean population visiting local clinics and hospitals. Among a total of 469,520 data sets of the lipid profile panel (total cholesterol, triglycerides, and high-density lipoprotein cholesterols), 142,932 lipid test sets with data on LDL-C and/or ApoB were used for statistical analysis. Using linear regression analysis, we created ApoB percentile value-derived LDL-C equations in a creating set and validated them with previously reported equations (a total of 11 equations) in comparison to directly measured LDL-C using two independent validating sets. Among all lipid test sets, the simultaneously measured ApoB test only accounted for 2.0%, indicating its underutilization in Korea. The ApoB-derived equations, which were derived in this study and previous studies, showed an overall agreement of ≥94.3% for NCEP ATP III criteria. However, the accuracy of the equations varied among data sets of populations. Future studies are needed to validate translational equations for ApoB and LDL-C in different populations to clarify the clinical implications of these equations.

## 1. Introduction

Cholesterol levels are a leading modifiable risk factor and the target of treatment for atherosclerotic cardiovascular diseases [1,2]. Historically, total cholesterol (TC), triglycerides (TG), LDL cholesterol (LDL-C), and high-density lipoprotein cholesterol (HDL-C) have been evaluated through lipid profile tests for the diagnosis and management of dyslipidemia and atherosclerotic cardiovascular diseases [3]. Apolipoprotein B (ApoB) is a key structural protein component of all major atherogenic lipoproteins such as chylomicron and its remnants, very low-density lipoprotein (VLDL), low-density lipoprotein (LDL), and lipoprotein (a); ApoB-containing lipoproteins retained in the arterial wall may provoke atherosclerotic cardiovascular diseases [4,5]. In the last decades, ApoB has been highlighted as a more accurate marker of cardiovascular risk than LDL-C and non-high-density lipoprotein cholesterol [4,6]. Recent European and Canadian guidelines have stated the superiority of ApoB over LDL-C [4]. Multi-ethnic studies of atherosclerosis confirmed ApoB as an independent risk factor for atherosclerosis [7]. While ApoB has been extensively studied in Europeans, its importance has been relatively less emphasized in Korea, and Korean guidelines for dyslipidemia suggest ApoB evaluation in patients with diabetes and familial hypercholesterolemia [8]. According to recent guidelines from the European Society of Cardiology (ESC) and the European Atherosclerosis Society (EAS), as well as the American College of Cardiology (ACC)/American Heart Association (AHA) guidelines and the Canadian Cardiovascular Society (CCS) guidelines, it is recommended to include ApoB measurements alongside the traditional lipid panel (TC, TG, HDL-C, and LDL-C) to define risk estimation and establish therapeutic targets for treatment [4,9,10,11]. However, the current Korean clinical guidelines primarily focus on traditional lipid tests, such as TC, TG, HDL-C, and LDL-C, and provide established cutoffs for risk estimation and therapeutic targets [8]. Although ApoB is mentioned in the guidelines, there is a lack of detailed, specific recommendations or guidelines regarding its interpretation [8].

The available data suggest that there are differences in the lipid profiles of Asians compared to Western populations, which can be attributed to a combination of genetic, environmental, and lifestyle factors [8,9,10,11,12,13]. For example, multi-ethnic studies have reported that Asians exhibit distinct dyslipidemia subtypes compared to non-Hispanic Whites, and even among different ethnicities within the Asian population, variations in lipid patterns exist [8,9,10,11,12,13]. It has been observed that Asians have a higher prevalence of elevated TG levels compared to non-Hispanic Whites, while Japanese men show a higher prevalence of low HDL-C levels compared to non-Hispanic White men [13]. However, no significant differences in low HDL-C were found between Korean men and non-Hispanic Whites [14]. Additionally, the study showed no significant differences in high LDL-C levels between Korean men and non-Hispanic Whites [13]. Therefore, conducting clinical studies to investigate lipid profiles in diverse ethnicities across various geographical regions is essential for effective dyslipidemia management on a global scale [9,10,11,12,13].

In addition, LDL-C is the primary target of lipid-lowering treatment in current clinical practice guidelines [4,14,15]. Because clinicians are more familiar with LDL-C concentrations and their cutoffs for treatment, in order to increase the use of ApoB, a translational tool to convert data to a familiar format report would be useful [14]. Cole et al. recently provided a simple equation to report ApoB levels measured in mass concentration units of mg/dL as well as in transformed LDL-C equivalent units (ApoB LDL-CEq) [14]. Cole et al. calculated ApoB LDL-CEq alongside the Friedewald (LDL-C_Friedewald) [16], Sampson (LDL-C_Sampson/NIH) [17], and Martin/Hopkins (LDL-C_Marin/Hopkins) equations [18]. However, these equations have not been validated in a large Korean population in multiple studies. Previous studies conducted in Korean populations have reported varying levels of agreement between equations for calculating LDL-C derived from different ethnic populations using various analytical methods and directly measured LDL-C [19].

Therefore, in this study, we aimed to investigate the utilization of the ApoB test in local clinics and hospitals in Korea and to develop and validate new translational equations for ApoB LDL-CEq in a large Korean population. In addition, the results of the proposed equations were compared with those of LDL-C_Friedewald, LDL-C_Sampson/NIH, LDL-C_Martin/Hopkins, LDL-C_Choi, and three equations by Cole et al. [14], as well as with the directly measured LDL-C (LDL-C_Direct).

## 2. Materials and Methods

### 2.1. Subjects

We retrospectively obtained anonymized clinical laboratory results for lipid profiles from the laboratory information system of Green Cross Laboratories between 1 January 2021 and 31 December 2021 for a population of Korean adults (age > 20 years) who visited local clinics and hospitals and underwent serum TG, TC, HDL-C, LDL-C, and ApoB testing. Results with TG greater than 1000 mg/dL (11 mmol/L) were excluded to minimize confounding factors, as extremely high TG levels can interfere with accurate measurements of other lipid parameters and may indicate specific pathological states affecting lipid parameters and patterns [14,20]. To ensure calculation reliability and minimize errors, results with HDL-C levels higher than TC were excluded. This exclusion was based on the understanding that a normal lipid profile typically exhibits TC calculated as the sum of HDL-C and non-HDL-C components [14,15,16,17,18,19,]. Because the aim of this study was to compare calculated LDL-C in comparison with LDL-C_Direct, test results with only TG, TC, or HDL-C without LDL-C_Direct or ApoB measurements were excluded.

### 2.2. Analytical Methods

Serum HDL-C, TG, TC, and LDL-C were measured using well-established automated Roche Cobas 8000 c702 analyzers (Roche, Mannheim, Germany) [14,19]. Serum ApoB was measured using an automated immunoturbidimetric assay using a Tina-quant Apolipoprotein B ver.2 reagent kit (Roche) on c702 analyzers (Roche). The analytical measurement range of the serum ApoB assay was 20.0–400.0 mg/dL. The calibrator of this assay was traceable to the IFCC reference material SP3-07 [21,22]. The manufacturer’s reference interval for ApoB in men ranged from 66.0 to 144.0 mg/dL and in women from 60.0 to 141.0 mg/dL. The accuracy of lipid measurements was assured through participation in accuracy-based external quality assurance programs by the Centers for Disease Control, USA; College of American Pathologists; and Korean External Quality Assessment Scheme. These quality assurance programs provide proficiency test materials with target values assigned by reference measurement methods. Participation in these quality assurance programs promotes standardization, validates testing methods, assesses proficiency, identifies errors and biases, and drives continuous improvement [23,24].

### 2.3. Definitions

In order to develop a translational equation for LDL-C based on ApoB in this study group (ApoB LDL-CEq_Choi), data sets from specimens with TC, TG, HDL, and ApoB data without LDL-C_Direct results were used (Creation Set). Linear regression analysis between calculated LDL using each equation (LDL-C_Sampson/NIH, LDL-C_Friedewald, LDL-C_Martin/Hopkins, and LDL-C_Choi) and ApoB percentile was performed [14].

Considering that the ApoB test has not been widely used in Korean patients, two independent data sets were used for validating calculated LDL-C; the first was for calculated LDL-C using equations derived from TC, TG, and HDL (LDL-C_Sampson/NIH, LDL-C_Friedewald, LDL-C_Martin/Hopkins, and LDL-C_Choi), and the second was for calculated LDL-C using all equations including the newly developed ApoB percentile-derived equation in this study population using the Creation Set (ApoB LDL-CEq_Sampson/NIH, ApoB LDL-CEq_Friedewald, ApoB LDL-CEq_Martin/Hopkins, and ApoB LDL-CEq_Choi) and equations previously reported by Cole et al. (Apo B LDL-CEq_Cole_Sampson/NIH, Apo B LDL-CEq_Cole_Friedewald, and Apo B LDL-CEq_Cole_Martin/Hopkins) [14].

In order to validate the calculated LDL-C in comparison with LDL-C_Direct for the first analysis, test results with TC, TG, HDL, and LDL-C_Direct without ApoB were defined as ‘Validation Set 1’. To validate the ApoB-derived equations, test results with TC, TG, HDL, LDL-C_Direct, and ApoB levels were defined as ‘Validation Set 2’.

Criteria for the National Cholesterol Education Program (NCEP) Adult Treatment Panel III (ATP III) for LDL-C were used to investigate the agreement of CVD risk categorization by equation as optimal LDL-C < 100 mg/dL, above optimal 100–129 mg/dL, borderline high 130–159 mg/dL, high 160–189 mg/dL, and very high ≥ 190 mg/dL [3].

### 2.4. Statistical Analysis

Non-parametric analysis was used when the data did not show a normal distribution (age and lipid results). Chi-square tests were used to compare to categorical variables (sex and NCEP ATP III criteria). Linear regression analysis was used to create equations to obtain LDL-C with ApoB results [14]. Calculated LDL-C using each equation was compared quantitatively with LDL-C_Direct using Bland–Altman plot analysis. The results were compared qualitatively based on agreement of NCEP ATP III categorization for LDL-C [19]. Systemic differences between calculated LDL-C and LDL-C_Direct levels were calculated as ‘calculated LDL-C minus LDL-C_Direct’. The percentage of systemic difference (%difference) was calculated as ‘calculated LDL-C and LDL-C_Direct/LDL-C_Direct × 100’. Absolute percentage error was calculated as 100 × absolute value [(y − ref)/ref], where ‘y’ is the observation and ‘ref’ is the reference value (LDL-C_Direct). Statistical analysis was executed using MedCalc Statistical Software Version 20.110 (MedCalc Software bv, Ostend, Belgium; https://www.medcalc.org; accessed on 29 March 2023). *p* values were considered significant at the 0.05 level.

### 2.5. Ethical Approval

This study was conducted according to the guidelines outlined in the Declaration of Helsinki, and all procedures involving human subjects were approved by the Institutional Review Board (IRB) of Green Cross Laboratories (GCL-2023-1010-01, 17 February 2023). A waiver of informed consent was approved by the IRB as the study was retrospective and involved no risk to subjects.

## 3. Results

### 3.1. Characteristics of Study Subjects and Lipid Results

**Figure 1:**
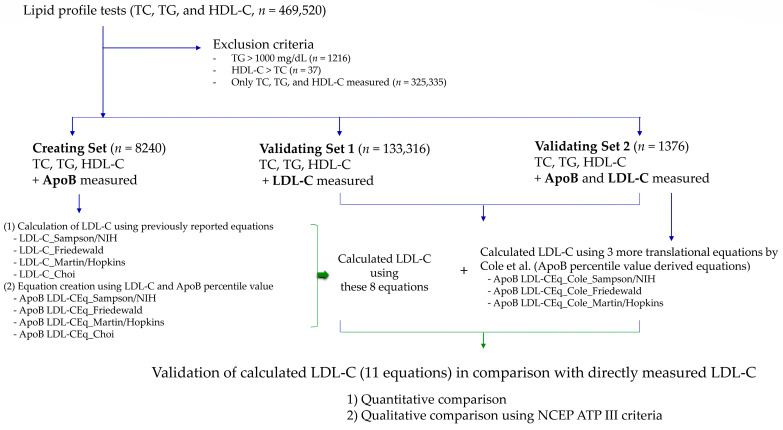
Study scheme. The development and validation of apolipoprotein B derived equations for calculating LDL-C were performed in comparison with previously reported equations by Cole et al. [14]. Abbreviations: ApoB, apolipoprotein B; HDL-C, high-density lipoprotein cholesterol; LDL-C, low-density lipoprotein cholesterol; TC, total cholesterol; TG, triglycerides.

Between 1 January 2021 and 31 December 2021, 469,520 Korean adults (213,637 men and 255,883 women) with a mean age of 55.6 years (SD 14.95) were tested for a lipid profile panel (TC, TG, and HDL-C). After applying exclusion criteria, 142,932 lipid test sets were used for statistical analysis; 8240 test sets for the Creation Set; 133,316 test sets for Validation Set 1; and 1376 test sets for Validation Set 2. The study scheme and baseline characteristics of each group of patients are summarized in Figure 1 and Table 1. The three datasets showed significant differences in age, sex distribution, and cholesterol levels except for LDL-C.

### 3.2. Equations for apoB LDL-CEq

Equations for ApoB LDL-CEq using linear regression analysis between the ApoB percentile of calculated LDL-C and the previously reported calculated LDL-C are summarized in Table 2. For regression analysis between the ApoB percentile and LDL-C level, 8240 patients with measurements of TC, TG, HDL-C, and ApoB without LDL-C_Direct were selected and categorized as the Creation Set.

### 3.3. Validating Equations for Calculated LDL-C

Equations for calculating LDL-C using the non-ApoB-derived percentile (LDL-C_Sampson/NIH, LDL-C_Friedewald, LDL-C_Martin/Hopkins, and LDL-C_Choi) were obtained from each data set. A comparison between the calculated LDL-C and LDL-C_Direct was performed for Validation Sets 1 and 2 (LDL-C_Direct was not available for the Creation Set).

**Figure 2:**
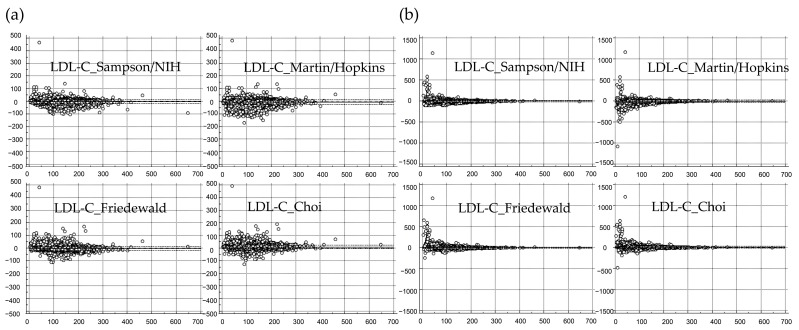
Comparison of calculated LDL-C using previously reported non-ApoB percentile-derived equations in comparison with directly measured LDL-C (LDL-C_Direct) in Validation Set 1 (*n* = 133,316). (**a**) Difference between LDL-C_Direct and calculated LDL-C. The *Y*-axis represents the difference between calculated LDL-C and directly measured LDL-C (mg/dL). (**b**) The %difference between LDL-C_Direct and calculated LDL-C using previously reported equations. The *Y*-axis represents the %difference between calculated LDL-C using each equation and LDL-C_Direct. The *X*-axis represents LDL-C_Direct. The maximum scale for the *Y*-axis is consistent across all plots, set at ±500 mg/dL for (**a**) and at ±1500% for the percentage difference in (**b**). The horizontal line represents the line of equality (with a difference of 0), while the dashed horizontal line represents the 95% confidence interval for the limits of agreement.

The systemic difference and %difference of the quantitative LDL-C level between LDL-C_Direct and the calculated LDL-C using each equation in Validation Set 1 are summarized using Bland–Altman plot analysis as in Figure 2.

In Validation Set 1 (*n* = 133,316), among four equations for the non-ApoB-derived value, LDL-C_Choi showed a higher LDL-C level than LDL-C_Direct with the largest mean difference (9.5 mg/dL, 95% CI 9.4 to 9.5) and mean %difference (9.7%, 95% CI 9.7 to 9.8). The LDL-C_Martin/Hopkins equation showed the smallest difference (−3.7 mg/dL, 95% CI −3.7 to −3.6) and %difference (−3.0%, 95% CI −3.0 to −2.9) from LDL-C_Direct, followed by LDL-C_Sampson/NIH (−4.3 mg/dL and −4.1%), LDL-C_Friedewald (−8.0 mg/dL and −8.0%), and LDL-C_Choi. For the absolute percentage error, LDL-C_Sampson/NIH showed the lowest (median 4.9%, 95% CI 4.8 to 4.9), followed by LDL-C_Martin/Hopkins (5.0%), LDL-C_Friedewald (6.5%), and LDL-C_Choi (8.9%). The calculated LDL-C values using some of these equations showed negative values, resulting in significant differences and percentage differences (Figure 2).

**Figure 3:**
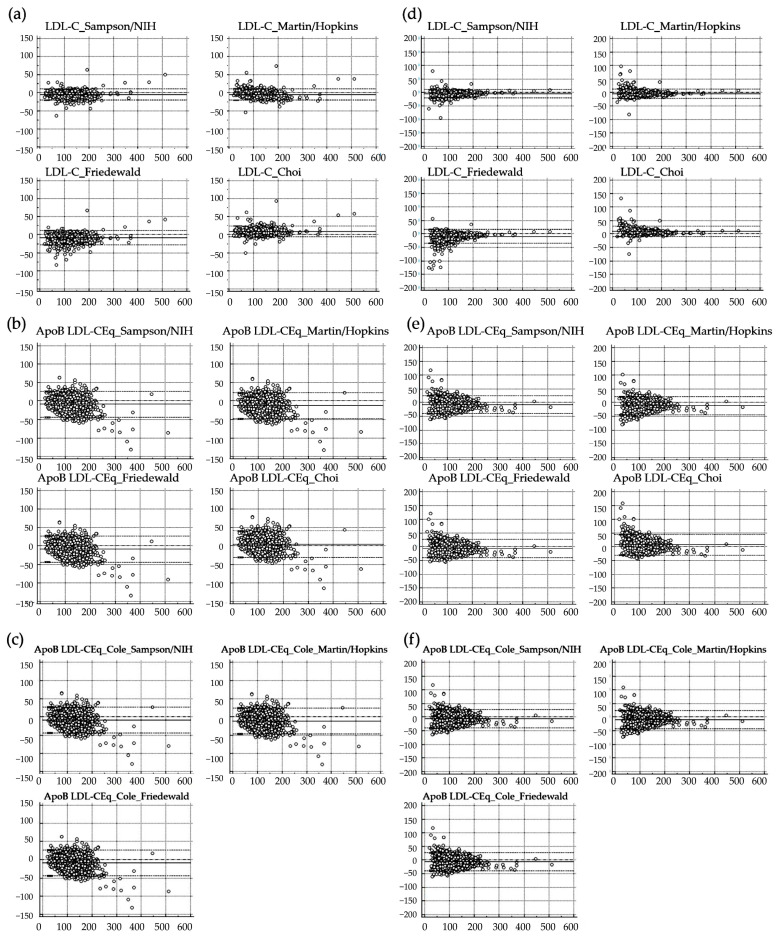
Comparison of calculated LDL-C with directly measured LDL-C (LDL-C_Direct) in Validation Set 2 (*n* = 1376). (**a**) Difference between LDL-C_Direct and calculated LDL-C using previously reported non-ApoB-derived equations. (**b**) Difference between LDL-C_Direct and calculated LDL-C using the proposed ApoB-derived equations. (**c**) Difference between LDL-C_Direct and calculated LDL-C using ApoB-derived equations by Cole et al. (**d**–**f**) The %difference data for (**a**–**c**). The *X*-axis represents LDL-C_Direct. The maximal value of the *Y*-axis is ±150 mg/dL for the difference from (**a**–**c**) and ±200% for the %difference from (**d**–**f**). The horizontal line represents the line of equality (with a difference of 0), while the dashed horizontal line represents the 95% confidence interval for the limits of agreement.

The systematic difference and %difference of quantitative LDL-C level between LDL-C_Direct and that of each equation in Validation Set 2 are summarized using Bland–Altman plot analysis as in Figure 3.

In Validation Set 2 (*n* = 1376), 11 equations for the calculated LDL-C were compared with LDL-C_Direct. Among these equations, the smallest systemic difference and %difference were observed for LDL-C_Martin/Hopkins (−4.7 mg/dL and 3.9%, respectively), followed by LDL-C_Sampson/NIH (−5.0 mg/dL and −4.9%). The LDL-C_Choi equation showed an 8.9 mg/dL mean systemic difference and a 9.3% difference. The equation of ApoB LDL-CEq_Choi showed a 5.1 mg/dL mean systemic difference and a 7.0% difference. The calculated LDL-C level was highest using LDL-C_Choi and ApoB LDL-CEq_Choi (positive mean systemic differences), while those using the other equations were lower than LDL-C_Direct (negative mean systemic differences).

Overall, ApoB percentile-derived equations showed higher absolute percentage errors than non-ApoB-derived equations. The maximum systemic difference and %difference were observed for ApoB LDL-CEq_Friedewald (−12.7 mg/dL and −11.3%, respectively), followed by ApoB LDL-CEq_Cole_Friedewald (−10.8 mg/dL and −9.4%). For the absolute percentage error, the lowest median error was observed for LDL-C_Martin/Hopkins (5.3%), followed by LDL-C_Sampson/NIH (5.6%). The highest median error was observed for ApoB LDL-CEq_Friedewald (14.1%) and then ApoB LDL-CEq_Cole_Friedewald (13.1%). Detailed results for the systemic difference, %difference, and absolute percentage error for a comparison of the calculated LDL-C and LDL-C_Direct are summarized in Supplementary Tables S1 and S2.

**Figure 4:**
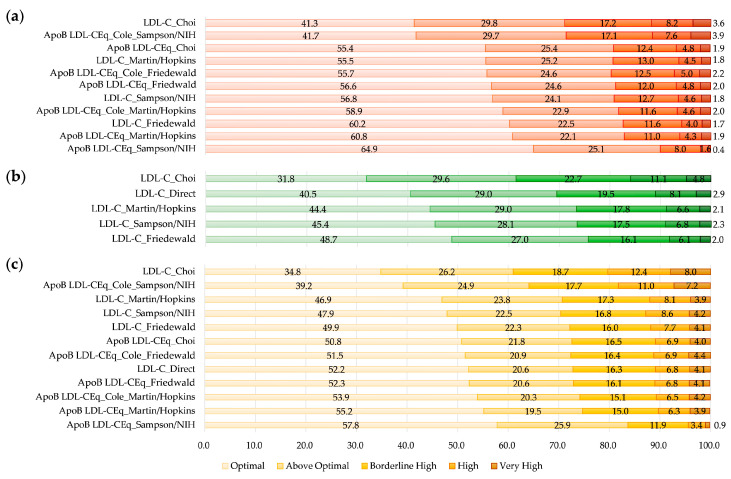
Proportion of data sets classified by NCEP ATP III Criteria for calculated LDL-C and directly measured LDL-C (%) in the Creation Set (**a**), Validation Set 1 (**b**), and Validation Set 2 (**c**). Darker colors indicate higher LDL-C levels.

Qualitative results using NCEP ATP III criteria were also compared among equations for LDL-C as summarized in Figure 4. The proportion of patients with optimal LDL-C was different among sets. Among the equations, LDL-C_Choi showed the smallest proportion of optimal LDL-C level for all data sets. Overall, the ApoB-derived equations had larger proportions of optimal LDL-C level than non-ApoB-derived equations except for the Friedewald equations for the Creation Set. Among all ApoB-derived equations, ApoB LDL-CEq_Sampson/NIH had the largest proportion of optimal LDL-C, followed by ApoB LDL-CEq_Martin/Hopkins. The proportion of patients with hyper-LDL-cholesterolemia based on NCEP ATP III criteria (LDL-C ≥ 160 mg/dL) was different among equations, ranging from 2.0% by LDL-CEq_Sampson/NIH to 11.8% by LDL-C_Choi in the Creation Set and from 4.3% by ApoB LDL-CEq_Sampson/NIH to 20.4% by LDL-C_Choi in Validation Set 2.

**Figure 5:**
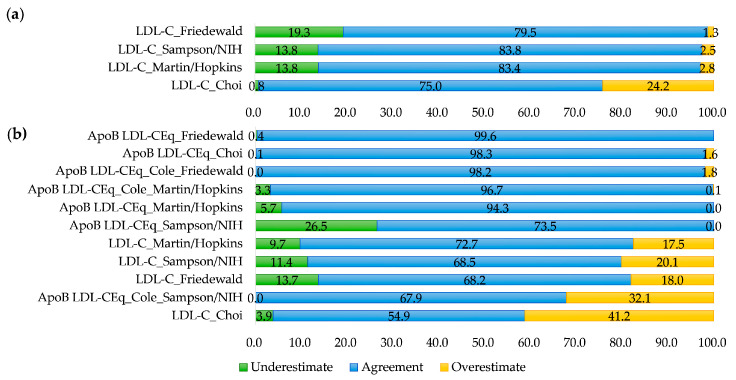
Proportion of agreement of NCEP ATP III criteria categorization for calculated LDL-C in comparison with directly measured LDL-C (LDL-C_Direct, %). (**a**) Validation Set 1 and (**b**) Validation Set 2. For creating sets, LDL-C_Direct was not measured and agreement could not be evaluated.

An agreement of NCEP ATP III criteria for LDL-C between the calculated LDL-C and LDL-C_Direct in the Validation Sets is summarized in Figure 5. In Validation Set 1, LDL-C_Sampson/NIH and LDL-C_Martin/Hopkins showed similar agreement with LDL-C_Direct (83.8% and 83.4%, respectively). In the same set, LDL-C_Choi showed an overestimation of LDL-C in comparison with LDL-C_Direct (24.2%).

In Validation Set 2, ApoB LDL-CEq_Friedewald showed the greatest agreement with LDL-C_Direct (99.6%), followed by ApoB LDL-CEq_Choi (98.3%). In Validation Set 2, ApoB LDL-CEq_Sampson/NIH showed an underestimation of NCEP ATP III criteria in comparison with LDL-C_Direct (26.5%). In Validating Set 2nd, ApoB-derived equations showed greater agreement for NCEP ATP III criteria with LDL-C_Direct than did non-ApoB-derived equations, except for ApoB LDL-CEq_Sampson/NIH (overestimation of 32.1%). Non-ApoB-derived equations showed an overestimation of NCEP ATP III categorization compared to LDL-C_Direct (range 17.5% to 41.2%) in Validation Set 2.

### 3.4. ApoB Levels to Predict LDL-C

Table 3 summarizes the ranges of ApoB levels, based on each ApoB-derived equation, for predicting optimal LDL-C levels (<100 mg/dL) and high LDL-C levels (≥160 mg/dL), as well as the ranges of LDL-C levels for predicting ApoB levels at or above the upper limit of the reference range for men (>144 mg/dL) and women (>141 mg/dL). Overall, if LDL-C levels were <160 mg/dL (the threshold for high LDL-C according to NCEP ATP III criteria), it appeared likely that ApoB levels would be <140 mg/dL (close to the upper limit of the reference interval for women).

## 4. Discussion

In this study, we proposed translational equations for ApoB and LDL-C levels and validated them using independent validation sets in comparison with previously reported equations to assess the calculated LDL-C level in a large Korean population who visited local clinics and hospitals.

In this study, the simultaneous measurement of LDL-C was performed in 28.7% of all tests, while ApoB testing was performed in only 2.0% of tests using the traditional lipid panel (TC, TG, and HDL-C). This demonstrates the underutilization of these tests in Korean patients visiting local clinics and hospitals. According to the Korean Guidelines for the Management of Dyslipidemia, ApoB measurements are recommended for diabetes patients [8]. According to the diabetes factsheet in Korea 2021, the age-standardized prevalence of hyper-LDL-cholesterolemia (LDL-C ≥ 160 mg/dL) in Korea is 76.5% in diabetes patients, and the prevalence of diabetes in 2021 was 16.7% among Korean adults aged 30 years or older. This indicates that 12.6% of clinical specimens could be calculated for ApoB [25]. According to the public database Healthcare Bigdata Hub by the Health Insurance Review & Assessment Service Korea (HIRA), 400,764 patients had apolipoprotein data but it did not differentiate between apolipoprotein A and ApoB [26] in 2021. This number was about 2.7% of all patients who underwent reimbursable TC tests, which were performed using an automated enzymatic assay in 2021 (14,990,233 patients, code D2611), confirming underutilization (2.0%). The use of the test could be improved with more clinical evidence to confirm its clinical implications in patient management in Korean populations and with appropriate educational programs targeted toward physicians [27].

In the present study, ApoB percentile-derived LDL-C equations showed comparable slope and intercept with those from equations provided by Cole et al. (Table 2) [14]. Overall, if ApoB levels are <140 mg/dL (close to the upper limit of the reference interval for women), LDL-C levels were predicted to be <160 mg/dL (the threshold for high LDL-C according to NCEP ATP III criteria). Clinicians can use this information to easily predict lipoprotein levels (Table 3). A translational tool for converting ApoB and LDL-C may promote the use of this test, as with the application of glycated hemoglobin and estimated average glucose level [14]. In the present study, ApoB percentile-derived equations in the Creation Set showed comparable results for qualitative NCEP ATP III LDL-C criteria to Validation Set 2. However, non-ApoB-derived equations in previously reported and well-validated equations including LDL-C_Sampson/NIH, LDL-C_Friedewald, and LDL-C_Martin/Hopkins showed underestimated and overestimated LDL-C levels in comparison with LDL-C_Direct in Validation Set 2. The characteristics of the populations were different among data sets except for LDL-C cholesterol levels. These results suggest that the performance of equations estimating LDL-C can be influenced by the characteristics of specific populations. In the same vein, the LDL-C_Choi equation derived using data from the same laboratory in a different study period showed overestimated LDL-C levels in comparison with LDL-C_Direct in the present study [19]. The LDL-C_Choi equation was not the best predictor for LDL-C in ApoB-measured groups in the same laboratory. These findings emphasize the importance of equation validation in different populations. Additional studies with detailed clinical information on lipid results and comorbidities affecting the accuracy of equations are needed.

The strength of this study was its large study population with lipid results from local clinics and hospitals. Considering that dyslipidemia is usually managed in such institutions, the study population of the present study included more real-world data than previous university-hospital-based studies [4,28]. In addition, the created and validated ApoB-derived LDL-C equation and previously reported equations provided robust results.

The limitations of this study include a lack of detailed clinical data about dyslipidemia, such as familial history, comorbidities, and lipid-lowering medications [4,19,29]. The physiological relevance of the prediction model is constrained by the limited availability of detailed clinical information, including data on cardiovascular disease endpoints such as heart attacks, emphasizing the necessity for further exploration in future research. The generalizability of this study may be limited to specific patients who visited local clinics and hospitals and had available LDL-C_Direct and ApoB measurements. Future studies based on detailed clinical information regarding dyslipidemia are needed to clarify the clinical implications of various equations estimating LDL-C and its relationship with ApoB. The cost–benefit analysis of incorporating an ApoB test into a traditional lipid panel analysis was not within the scope of this study. However, considering that ApoB has been suggested as a risk enhancer and therapeutic target for treatment in international guidelines, future studies should investigate the cost–benefit of the ApoB test along with its clinical utility [2,4,10,12,30,31].

## 5. Conclusions

In conclusion, we investigated the utilization of the ApoB test in local clinics and hospitals in Korea and developed and validated new translational equations for ApoB LDL-CEq in a large Korean population along with other previously reported equations for calculated LDL-C in comparison with LDL-C_Direct. The created ApoB-derived LDL-C equations (ApoB LDL-CEq) in this study showed comparable results with previously reported ApoB-derived equations as a translational tool. The results of this study will expand basic knowledge about equations for LDL-C and predictive changes in the prevalence of LDL-C according to the equation used. Considering that the accuracy of the equations varied by population set, future studies are needed to validate their accuracy and performance with detailed clinical information.

## Figures and Tables

**Table 1:** Baseline characteristics.

Characteristics	Total (*n* = 142,932)	Creating Set (*n* = 8240)	Validating Set 1st (*n* = 133,316)	Validating Set 2nd (*n* = 1376)	*p*-Value
Analytes	Sum of all data sets	TC, TG, HDL-C, ApoB	TC, TG, HDL-C, LDL-C	TC, TG, HDL-C, ApoB, LDL-C	
Age, years (median, IQR)	54.6 (44.2 to 64.1)	59.3 (48.4 to 67.2)	54.3 (44.0 to 63.9)	54.5 (42.8 to 64.5)	<0.0001
Sex (*n*, %)					<0.0001
Male	68,240 (47.7%)	3869 (47.0%)	63,801 (47.9%)	570 (41.4%)	
Female	74,692 (52.3)	4371 (53.0%)	69,515 (52.1%)	806 (58.6%)	
Lipid test results, mg/dL (median, IQR)					
TC	185.0 (157.0 to 215.0)	175.0 (150.0 to 205.0)	186.0 (157.0 to 215.0)	187.0 (157.0 to 221.5)	<0.0001
TG	120.0 (84.0 to 178.0)	123.0 (87.0 to 182.0)	120.0 (84.0 to 177.0)	114.0 (77.5 to 174.0)	<0.0001
HDL-C	53.0 (44.0 to 63.0)	53.0 (44.0 to 63.0)	53.0 (44.0 to 63.0)	56.0 (44.6 to 67.0)	<0.0001
Non-HDL-C	129.0 (102.0 to 160.0)	119.0 (95.0 to 150.0)	129.0 (102.0 to 160.0)	127.0 (98.0 to 164.0)	<0.0001
LDL-C ^1^	109 (84.0 to 136.0)	-	109.0 (84.0 to 136.0)	108.0 (81.0 to 142.0)	NS
ApoB ^1^	89.6 (73.5 to 110.6)	89.1 (73.5 to 109.6)	-	92.2 (74.1 to 118.8)	<0.0001

^1^ The total number of LDL-C measurements was 134,692 and the total number of ApoB measurements was 9616. Abbreviations: ApoB, apolipoprotein B; HDL-C, high-density lipoprotein cholesterol; IQR, interquartile range; LDL-C, low-density lipoprotein cholesterol; Non-HDL-C, non-HDL cholesterol calculated from TC minus HDL-C; NS, not significant; TC, total cholesterol; TG, triglycerides.

**Table 2:** Equations for calculated LDL-C.

Abbreviation of Equation	Equation Driven	Equation (y) = LDL-C
LDL-C_Sampson/NIH	By Sampson et al. [17]	y = (TC/0.948) − (HDL-C/0.971) − [(TG/8.56) + {(TG × Non-HDL-C)/2140} − (TG)^2^/16100] − 9.44
LDL-C_Friedewald	By Friedewald et al. [16]	y = TC − HDL-C − (TG/5)
LDL-C_Martin/Hopkins	By Martin et al. [18]	y = TC − HDL-C − (TG/different adjustable factors)
LDL-C_Choi	By Choi et al. [19]	y = TC − 0.87 × HDL-C − 0.13 × TG
ApoB LDL-CEq_Sampson/NIH	This study	y = 1.352 × (ApoB) − 27.163
ApoB LDL-CEq_Friedewald	This study	y = 1.382 × (ApoB) − 33.756
ApoB LDL-CEq_Martin/Hopkins	This study	y = 1.327 × (ApoB) − 23.669
ApoB LDL-CEq_Choi	This study	y = 1.393 × (ApoB) − 17.113
ApoB LDL-CEq_Cole_Sampson/NIH	By Cole et al. [14]	y = 1.38 × (ApoB) – 29
ApoB LDL-CEq_Cole_Friedewald	By Cole et al. [14]	y = 1.385 × (ApoB) − 32.2
ApoB LDL-CEq_Cole_Martin/Hopkins	By Cole et al. [14]	y = 1.348 × (ApoB) − 26.4

Unit for lipoprotein is mg/dL.

**Table 3:** Apolipoprotein B levels to predict LDL-C.

Abbreviation of Equation	Equation Driven	Equation (y) = LDL-C	ApoB Levels for LDL-C Target		LDL-C Levels for ApoB Level	
			100	160	141 ^1^	144 ^2^
ApoB LDL-CEq_Sampson/NIH	This study	y = 1.352 × (ApoB) − 27.163	94	138	163	168
ApoB LDL-CEq_Friedewald	This study	y = 1.382 × (ApoB) − 33.756	97	140	161	165
ApoB LDL-CEq_Martin/Hopkins	This study	y = 1.327 × (ApoB) − 23.669	93	138	163	167
ApoB LDL-CEq_Choi	This study	y = 1.393 × (ApoB) − 17.113	84	127	179	183
ApoB LDL-CEq_Cole_Sampson/NIH	By Cole et al. [14]	y = 1.38 × (ApoB) − 29	94	137	166	170
ApoB LDL-CEq_Cole_Friedewald	By Cole et al. [14]	y = 1.385 × (ApoB) − 32.2	96	139	163	167
ApoB LDL-CEq_Cole_Martin/Hopkins	By Cole et al. [14]	y = 1.348 × (ApoB) − 26.4	94	138	164	168

^1^ Upper limit of reference interval for women; ^2^ Upper limit of reference interval for men. Unit for lipoprotein is mg/dL.

## Data Availability

The datasets generated and analyzed during the current study are available from the corresponding authors on reasonable request.

## Supplementary Materials

The following supporting information can be downloaded at: https://www.mdpi.com/article/10.3390/nu15122786/s1, Table S1: Comparison of calculated LDL-C Levels with directly measured LDL-C in the Validating Set 1 (*n* = 133,316); Table S2: Comparison of calculated LDL-C levels with directly measured LDL-C in Validating Set 2nd (*n* = 1376).
