# Supplementary material for: Development and Validation of Multiple Equations for Low-Density Lipoprotein and Apolipoprotein B in Korean Patients Visiting Local Clinics and Hospitals
Source: Nutrients. 2023 Jun 17;15(12):2786. doi: 10.3390/nu15122786 (PMC10301897; doi:10.3390/nu15122786)
Supplement: Supplementary file 1 [file nutrients-15-02786-s001.zip › nutrients-2397433-supplementary.pdf]

Supplementary Table S1. Comparison of calculated LDL-C Levels with directly measured LDL-C in the Validating Set 1 (n=133,316)

**Comparison and equations**	**Data**	
Systemic differences, mg/dL [1]	Mean (SD)	95% confidence interval
LDL-C_Sampson/NIH	-4.3 (7.83)	-4.34 to -4.26
LDL-C_Friedewald	-8.0 (10.02)	-8.01 to -7.90
LDL-C_Martin/Hopkins	-3.7 (7.88)	-3.70 to -3.61
LDL-C_Choi	9.5 (7.59)	9.44 to 9.52
Systemic differences, % [2]	Mean (SD)	95% CI
LDL-C_Sampson/NIH	-4.1 (9.32)	-4.12 to -4.02
LDL-C_Friedewald	-8.0 (13.35)	-8.10 to -7.96
LDL-C_Martin/Hopkins	-3.0 (10.31)	-3.01 to -2.90
LDL-C_Choi	9.76 (10.83)	9.70 to 9.82
Absolute percentage error, %	Median	95%CI
LDL-C_Sampson/NIH	4.9	4.82 to 4.88
LDL-C_Friedewald	6.5	6.48 to 6.57
LDL-C_Martin/Hopkins	5.0	5.00 to 5.06
LDL-C_Choi	8.9	8.80 to 8.89

[1] calculated LDL-C minus directly measured LDL-C. [2] calculated LDL-C minus directly measured LDL-C) / directly measured x 100

Supplementary Table S2. Comparison of calculated LDL-C levels with directly measured LDL-C in Validating Set 2nd (n=1376)

Comparison and equations	Data	
Systemic differences, mg/dL [1]	Mean (SD)	95% confidence interval
LDL-C_Sampson/NIH	-5.0 (7.95)	-5.42 to -4.58
LDL-C_Friedewald	-8.6 (10.02)	-9.15 to -8.09
LDL-C_Martin/Hopkins	-4.7 (8.25)	-5.09 to -4.22
LDL-C_Choi	8.9 (7.86)	8.50 to 9.33
ApoB LDL-CEq_Sampson/NIH	-9.0 (18.03)	-9.97 to -8.06
ApoB LDL-CEq_Friedwald	-12.7 (18.10)	-13.63 to -11.72
ApoB LDL-CEq_Martin/Hopkins	-8.0 (18.01)	-8.93 to -7.02
ApoB LDL-CEq_Choi	5.1 (18.15)	4.10 to 6.02
ApoB LDL-CEq_Cole_Sampson/NIH	-8.1 (18.12)	-9.06 to -7.14
ApoB LDL-CEq_Cole_Friedwald	-10.8 (18.12)	-11.77 to -9.86
ApoB LDL-CEq_Cole_Martin/Hopkins	-8.6 (18.03)	-9.60 to -7.69
Systemic differences, % [2]	Mean (SD)	95% confidence interval
LDL-C_Sampson/NIH	-4.9 (8.18)	-5.32 to -4.46
LDL-C_Friedewald	-9.0 (12.90)	-9.64 to -8.28
LDL-C_Martin/Hopkins	-3.9 (8.95)	-4.33 to -3.38
LDL-C_Choi	9.27 (9.66)	8.76 to 9.78
ApoB LDL-CEq_Sampson/NIH	-7.1 (16.81)	-8.01 to -6.23
ApoB LDL-CEq_Friedwald	-11.3 (16.99)	-12.21 to -10.42
ApoB LDL-CEq_Martin/Hopkins	-5.7 (16.80)	-6.61 to -4.84
ApoB LDL-CEq_Choi	7.0 (18.92)	6.03 to 8.03
ApoB LDL-CEq_Cole_Sampson/NIH	-6.5 (17.10)	-7.43 to -5.6
ApoB LDL-CEq_Cole_Friedwald	-9.4 (17.03)	-10.31 to -8.51
ApoB LDL-CEq_Cole_Martin/Hopkins	-6.7 (16.84)	-7.58 to -5.80
Absolute percentage error, %	Median	95% confidence interval
LDL-C_Sampson/NIH	5.3	4.92 to 5.66
LDL-C_Friedewald	7.0	6.48 to 7.45
LDL-C_Martin/Hopkins	5.6	5.20 to 6.00
LDL-C_Choi	8.4	7.90 to 8.86
ApoB LDL-CEq_Sampson/NIH	11.9	11.11 to 12.66
ApoB LDL-CEq_Friedwald	14.1	13.23 to 14.99
ApoB LDL-CEq_Martin/Hopkins	11.0	10.53 to 11.90
ApoB LDL-CEq_Choi	10.3	9.57 to 11.10
ApoB LDL-CEq_Cole_Sampson/NIH	11.6	10.84 to 12.28
ApoB LDL-CEq_Cole_Friedwald	13.1	12.34 to 13.84
ApoB LDL-CEq_Cole_Martin/Hopkins	11.6	10.87 to 12.36

[1] calculated LDL-C minus directly measured LDL-C. [2] calculated LDL-C minus directly measured LDL-C) / directly measured x 100
